# Factors Associated with Suicide and Bankruptcy in Japanese Pathological Gamblers

**DOI:** 10.1007/s11469-014-9492-3

**Published:** 2014-03-19

**Authors:** Yasunobo Komoto

**Affiliations:** 1 Kurihama Medical and Addiction Center, 5-3-1, Nobi, Yokosuka City, Japan; 2 Okayama Psychiatric Medical Center, Okayama, Japan

**Keywords:** Pathological gambling, Suicide, Personal bankruptcy, Family history of addiction, Psychiatric complications

## Abstract

Pathological gambling can lead to a number of deleterious consequences, including bankruptcy and suicide. The present study examined the correlation between clinical characteristics of pathological gamblers and history of bankruptcy and suicide attempts. Subjects (141; 124 male) were outpatients at a psychiatric hospital from 2007 to 2010. Demographic and medical variables including age, gender, age of gambling onset, psychiatric complications, suicide attempts, and bankruptcy were assessed. Positive correlations were observed between suicide attempt and female gender, family history of addiction, and unemployment (*p* < 0.05). Similar variables correlated with bankruptcy (*p* < 0.05). Multivariate analysis revealed that a family history of addiction was the strongest independent predictor of suicide and bankruptcy. Results suggest that treatment for pathological gambling should address the patient’s past experience with addict family members, especially if the patient reports a history of bankruptcy or suicidal ideation.

Pathological gambling is a significant public health problem characterized by persistent and recurrent maladaptive gambling patterns. It is associated with impaired functioning, reduced quality of life, and mental illness (Hodgins et al. 2011). The prevalence rate of pathological gambling (0.2–5.3 % worldwide) varies according to its defining parameters and the availability and accessibility of gambling activities (Wardle et al. 2007). Generally, suicide attempts and bankruptcy are the most severe consequences and important indicators of more extreme variants in the progress of pathological gambling (Grant et al. 2010; Lee et al. 2011; Petry and Kiluk 2002); these consequences indicate psychological and social severity, respectively.

As findings indicate that 17.0–39.5 % of treatment-seeking pathological gamblers attempt suicide, a number of studies have explored the relationship between these two factors (Hodgins et al. 2006; Kausch 2003; Ledgerwood et al. 2005; Petry and Kiluk 2002; Tang et al. 2007). Gamblers who have considered or attempted suicide have a greater number of psychiatric complications, especially depression and substance abuse disorders. Relative to non-suicidal gamblers, evidence suggests that suicidal gamblers begin gambling at an earlier age, have greater debt, are more likely to experience marital difficulties, have participated in illegal behaviors, and have family histories of addiction. Further, female gamblers are more likely to consider suicide. The rate of bankruptcy is also substantially higher in pathological gamblers, with approximately 17.0–18.0 % of pathological gamblers having a history of bankruptcy declaration. Despite this, there is minimal research on the relationship between pathological gambling and personal bankruptcy (Grant et al. 2010; Petry and Kiluk 2002). These studies suggest that gamblers who file for bankruptcy are more likely to be unmarried, have an early onset of problem gambling, diagnosed with depressive and substance abuse disorders, and have a first-degree relative or family member with an alcohol-abuse disorder (Grant et al. 2010).

Gambling opportunities are abundant in Japan; it is therefore common: an estimated 5.5 % of adults are pathological gamblers (defined by a score ≥5 on the South Oaks Gambling Screen) (Higuchi 2008). Non-strategic gambling activities, such as Pachinko and slot machines, are most popular. Also, in Japan, suicide attempts and personal bankruptcy are the most severe consequences of pathological gambling (Tanabe 2010). Prevention of these consequences requires an understanding of the differences between progressive and non-progressive gambling, as well as the factors associated with suicide and bankruptcy. The purpose of this study was to examine the clinical characteristics associated with suicide and bankruptcy in treatment-seeking pathological gamblers in a Japanese psychiatric hospital and to identify the specific risk factors that may contribute to the progress of pathological gambling.

## Materials and Methods

### Participants

A retrospective cross-sectional design using data from medical records was employed in the current study. Participants were 141 adult outpatients, primarily diagnosed as pathological gamblers, of the addiction recovery unit of a psychiatric hospital from March 2007 to February 2010. Diagnostic criteria were derived from the DSM-IV-TR (American Psychiatric Association 2000). Patients with a history of a psychotic episode characterized by positive symptoms and manic states were excluded from the study. This investigation was carried out in accordance with the Declaration of Helsinki, and approved by the ethical committee of Okayama Psychiatric Medical Center.

### Procedure

Demographic and medical variables were retrospectively obtained from medical records. All examined variables were based on the participant’s first hospital visit. Demographic variables included gender, age, marital status, family history of addiction (defined as a second-degree or closer family member with an addiction disorder), education (greater or less than 12 years of education), and employment status. Medical variables included the age of gambling onset, age at which problem gambling first surfaced, time lag between gambling onset and the onset of problematic gambling (time lag), age at first addiction-related hospital visit, duration of problem gambling, predominant gambling activity (non-strategic or not), current psychiatric complications, number of lifetime suicide attempts, total amount of debt, and method of debt management (bankruptcy or not).

### Statistical Analysis

Data entry and statistical analysis were performed using Microsoft Excel® 2010. A Pearson correlation analysis was used to assess correlations between behavioral markers of severity (suicide attempts and bankruptcy) and other variables. To identify the independent predictors of attempted suicide and bankruptcy, multiple stepwise backward linear regression analysis was used and an analysis of variance for the full regression models was performed. Statistical significance was set at *p* < 0.05.

## Results

Data from the medical records of 141 pathological gamblers were analyzed; Table 1 summarizes participant characteristics. The mean age of subjects was 44.6 years (SD = 12.4), 87.9 % were male, 41.8 % had completed over 12 years of education, 59.6 % were married, 77.3 % were employed, 10.6 % had a family history of addiction, 7.86 % had a family history of gambling addiction, and 26.2 % had psychiatric complications. Psychiatric complications included mood disorders (10.6 %), schizophrenia (4.24 %), neurotic (obsessive or dissociative) disorders (3.54 %), autism spectrum disorders (2.83 %), substance abuse disorders (2.83 %), and kleptomania (1.41 %). One subject’s pathological gambling was presumed to result from anti-Parkinsonism drugs. Mood disorders (*n* = 15) consisted of three cases of depression with somatic symptoms (endogenomorphic type), seven cases of depression without somatic symptoms (non-endogenomorphic type), and five cases of bipolar disorder (Table 2). The mean age of gambling onset was 21.3 years (SD = 6.61), and the mean age at which problem gambling surfaced was 32.3 years (SD = 10.4). The time lag between gambling onset and problematic gambling was 10.9 years (SD = 8.82). The mean age at which subjects first visited the hospital for an addiction-related problem was 42.6 years (SD = 12.4). The mean duration of problem gambling was 12.3 years (SD = 8.17). The most prevalent gambling activities were Pachinko or slot machines (89.4 %). The average amount of debt was 6,128,000 yen. Finally, 12.1 % had a history of attempted suicide, and 10.6 % had a history of bankruptcy.

**Table 1:** Description of subjects (*n* = 141)

Gender: Male (%)	124 (87.9)
Age: Average (SD)	44.6 (12.4)
Married (%)	84 (59.6)
Educated beyond 12 years (%)	59 (41.8)
Employed (%)	109 (77.3)
Addiction-related family history (%)	15 (10.6)
(Gambling related) (%)	11 (7.86)
Psychiatric complications (%)	37 (26.2)
Age of gambling onset: average (SD)	21.3 (6.61)
Age of problem-gambling onset: average (SD)	32.3 (10.4)
Time lag: average (SD)	10.9 (8.82)
Age at first hospital visit: average (SD)	42.6 (12.4)
Duration of gambling problem (years): average (SD)	12.3 (8.17)
Primary gambling activity: Pachinko or slot machines (%)	126 (89.4)
Total amount of debt(thousand yen): average (SD)	6128 (8032)
Attempt at suicide (%)	17 (12.1)
Personal bankruptcy (%)	15 (10.6)

**Table 2:** Psychiatric complications (*n* = 37)

Mood disorder	15
Depression with somatic symptoms	3
Depression without somatic symptoms	7
Bipolar disorder	5
Schizophrenia	6
Neurotic disorder	5
Autism spectrum disorder	4
Substance abuse disorder	4
Other addiction disorder	2
Induced by anti-Parkinsonism drugs	1

Attempted suicide and bankruptcy were significantly correlated with a number of the examined variables (Table 3). Attempted suicide was positively correlated with female gender (*p* < 0.01), family history of addiction (*p* < 0.01), and unemployment (*p* < 0.05). Bankruptcy was positively correlated with female gender (*p* < 0.05), family history of addiction (*p* < 0.05), unemployment (*p* < 0.01), and unmarried marital status (*p* < 0.05).

**Table 3:**
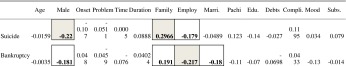
Correlations^a^ between suicide attempts, bankruptcy, and other variables (demographic and medical variables)

statistically significant at *p* < 0.05

statistically significant at *p* < 0.01

^a^values represent Pearson’s correlation coefficients

Multivariate analysis (Tables 4 and 5) indicated that a family history of addiction was most predictive of suicide attempts and bankruptcy. The standardized coefficient showed that unemployment was an additional predictor of bankruptcy. However, these variables accounted for only 6.8 % (bankruptcy) and 8.9 % (attempted suicide) of the variability in pathological gamblers.

**Table 4:** Best-fitting linear regression model for suicide attempts in pathological gamblers

	Unstandardized coefficients		Standardized coefficients	*t*-test	*p*-value
	B	Std. error			
Constant	0.10008	0.03092		3.2609	0.0014
Family	0.2992	0.08152	0.2992	3.6696	0.000034

r-squared = 0.08949; Model ANOVA: *F* = 13.46, *p* = 0.000346

**Table 5:** Best-fitting linear regression model for bankruptcy in pathological gamblers

	Unstandardized coefficients		Standardized coefficients	*t*-test	*p*-value
	B	Std. error			
Constant	0.25118	0.07478		3.3585	0.001016
Family	0.18129	0.08116	0.18522	2.2336	0.02714
Employment	−0.16559	0.07954	−0.17263	2.0817	0.03923

r-squared = 0.06769; Model ANOVA: *F* = 4.9374, *p* = 0.008511

## Discussion

The present study compared the clinical characteristics of treatment-seeking pathological gamblers with and without a history of attempted suicide or personal bankruptcy, two severe consequences of pathological gambling. Suicide attempt and bankruptcy were significantly associated with female gender, family history of addiction, and unemployment; thus, these variables were common risk factors predictive of suicide attempt or bankruptcy. Results additionally revealed a positive correlation between bankruptcy and unmarried status. Similarly, one report suggests an association between bankruptcy and divorce or separation (Caputo 2008). The present study further demonstrated through multivariate analysis that a family history of addiction was a greater predictor of suicide attempt and bankruptcy than female gender and unemployment. Interestingly, attempted suicide and bankruptcy were not significantly associated with each other; however, the independence of these events may suggest that each represents one side of a gambling-consequence continuum. Regardless, it is unsurprising that one of the most influential factors in the severity and prognosis of pathological gambling is a family history of addiction.

A maladaptive family member with an addiction disorder may engender maladaptive social and emotional coping skills in other family members (Bijttebier and Goethals 2006). The dominant aspect of these coping skills may be an impulsive coping strategy (Acheson et al. 2011). This impulsive coping strategy and a poor ability to seek help are associated with shame, which usually coincides with resentment towards the addicted family member (Yi and Kanetkar 2011). Further, these coping strategies often facilitate the development of pathological gambling and result in suicide attempts and/or bankruptcy. For example, psychodynamic theorists hypothesize that some people use gambling to cope with self-punitive, harsh superegos and to decrease unconscious stress levels (Sadock et al. 2009). Developed in Japan, insight-oriented Naikan therapy has been shown to improve subject cognition and self-esteem by asking the subject to recall an instance of parental (or caregiver) love (Komoto 2013; Maeshiro 2009). Therefore, the psychotherapeutic strategy for pathological gamblers with a family history of addiction should address issues of poor self-esteem and shame. Further, pathological gamblers who have not experienced personal bankruptcy but have a family history of addiction should be carefully evaluated for suicidal ideation.

The prevalence rates of attempted suicide (12.1 %) and bankruptcy (10.6 %) in the present study were not greater than those previously reported (Grant et al. 2010; Hodgins et al. 2006; Kausch 2003; Ledgerwood et al. 2005; Meltzer et al. 2011; Petry and Kiluk 2002; Tang et al. 2007). The main finding, the association between a family history of addiction and suicide attempt or bankruptcy, is consistent with the prevailing literature on pathological gambling. However, the factor most frequently reported in previous literature is psychiatric complications, especially mood and substance abuse disorders. I speculate that suicide attempt and bankruptcy were not associated with mood and substance abuse disorders in the present study because of the low rate of complicated mood disorders and substance abuse disorders found in Japanese pathological gamblers. The ccomplication rate of substance abuse disorders in this study was 2.83 %, which is substantially less than that found in other countries (12.0–57.5 %) (Grant et al. 2010; Lee et al. 2011; Lorains et al. 2011). Further, the complication rate of mood disorders was also less that that reported in previous studies (10.8 % vs. 14.7–37.9 %) (Grant et al. 2010; Lee et al. 2011; Lorains et al. 2011). Therefore, the low prevalence of psychiatric complications, especially substance abuse disorders, in Japanese pathological gamblers might delay the progression of gambling frequency and consequently temper the risk of suicide and/or bankruptcy.

## Limitations

Several limitations in the present study should be addressed. First, this sample consisted of pathological gamblers who sought treatment in a psychiatric hospital; this might reflect a different character pattern than that of individuals who do not seek professional help for this disorder. Second, suicide attempt and bankruptcy were reported by the participants but not confirmed by any outside source. Therefore, the data used in this study were not entirely objective. Third, the data were derived from patients of only one hospital over the course of 3 years, without control groups. Although this introduced some bias, this homogeneity may have lessened the effect of other potential confounds such as environmental factors. Finally, additional scales or semi-structured interviews were not used to assess the severity of comorbid Axis I disorders; thus, the results must be interpreted with caution. Nevertheless, to my knowledge, this is the first cross-sectional study to investigate the clinical characteristics of pathological gambling associated with attempted suicide and bankruptcy in Japan.

## Conclusion

The present study explored the associations between characteristics of Japanese pathological gamblers and severe consequences of pathological gambling, specifically suicide attempt and personal bankruptcy. Results suggest that a family history of addiction strongly influences the prognosis and severity of pathological gambling. Assessing and managing past aversive experiences associated with addict family members should be a primary goal in the treatment of pathological gamblers, especially those with a history of suicide attempt or bankruptcy. Further research is necessary in order to confirm these findings.

In conclusion, the relationship between pathological gambling and culture should be addressed. In Japan, Pachinko and slot machine gambling are regarded by law as leisure activities and are essentially free from public control, as gambling is spreading broadly. This may make pathological gambling more prevalent in Japan relative to other countries. Therefore, in Japan, the popularity of pathological gambling might result in a low rate of psychiatric complications, and the majority of pathological gamblers may never endure severe consequences, such as suicide and bankruptcy. This differential effect on the development of pathological gambling has rarely been investigated and should be addressed in future studies.
